# Editorial: E-waste and heavy metals: health hazards and environmental impact

**DOI:** 10.3389/fpubh.2024.1506438

**Published:** 2024-11-15

**Authors:** Baby Tabassum

**Affiliations:** ^1^Department of Zoology, Toxicology Lab, Govt. Raza P.G. College, Rampur, Uttar Pradesh, India; ^2^Mahatma Jyotiba Phule Rohilkhand University, Bareilly, India

**Keywords:** e-waste, heavy metal, environmental distribution, exposure, human health and disease

As the topic editor for “*E-Waste and Heavy Metals: Health Hazards and Environmental Impact*,” I am honored to present a Research Topic that addresses the urgent global challenge of e-waste management and the health risks posed by heavy metals. In an era of rapid technological growth, e-waste has become a critical environmental concern, threatening both ecosystems and human health due to hazardous metals like lead, mercury, cadmium, and nickel.

This Research Topic integrates diverse studies that analyse the health consequences of heavy metals from a variety of perspectives. A cross-sectional study on the association between anxiety and blood cadmium, lead, and mercury is a notable manuscript that provides valuable perspectives into the neurotoxic effects of heavy metal exposure. Investigations on systemic inflammation due to combined heavy metals and the risk of metabolic disorders like metabolic syndrome and fatty liver disease are equally significant.

Moreover, pioneering investigations into remediation techniques, including the phytoremediation capabilities of earthworms in cadmium-polluted soil and revelations regarding mercury toxicity from conventional Ayurvedic methods, present promising avenues for alleviating the environmental and health consequences of heavy metal exposure. These studies, despite their diverse focus, converge on a singular theme: the pressing necessity for global consciousness and intervention to address waste management and mitigate heavy metal pollution.

Key insights and strategies:

- **Health implications:** the accepted studies demonstrate alarming associations between heavy metal exposure and a variety of health issues, emphasizing the necessity of immediate action.
- **Sustainable practices:** advocating for eco-friendly design and accountable recycling initiatives is crucial to alleviate the effects of e-waste on health and the environment.
- **Collaborative endeavors:** involving stakeholders—from producers to consumers—is essential for formulating efficient e-waste management strategies.

I express my profound gratitude to all authors and reviewers for their significant contributions to this Research Topic. The findings presented here not only deepen our scientific understanding but also emphasize the need for sustainable solutions in the management of e-waste and heavy metals to safeguard both public health and the environment.

I hope this Research Topic will serve as a significant step toward raising awareness, advancing research, and informing policies that can help mitigate the risks posed by these environmental hazards.

